# Correlation Between Decreased Amygdala Subnuclei Volumes and Impaired Cognitive Functions in Pediatric Bipolar Disorder

**DOI:** 10.3389/fpsyt.2020.00612

**Published:** 2020-06-26

**Authors:** Dong Cui, Yongxin Guo, Weifang Cao, Weijia Gao, Jianfeng Qiu, Linyan Su, Qing Jiao, Guangming Lu

**Affiliations:** ^1^ College of Radiology, Shandong First Medical University (Shandong Academy of Medical Sciences), Taian, China; ^2^ Collaborative Innovation Center of Magnetic Resonance Imaging of Brain Disease, Shandong First Medical University, Shandong Academy of MedicalSciences, Taian, China; ^3^ Department of Child Psychology, The Children’s Hospital, Zhejiang University School of Medicine, Hangzhou, China; ^4^ Mental Health Institute of The Second Xiangya Hospital, Central South University, Changsha, China; ^5^ Department of Medical Imaging, Jinling Hospital, Nanjing University School of Medicine, Nanjing, China

**Keywords:** pediatric bipolar disorder, mania, euthymia, amygdala subnuclei, magnetic resonance imaging

## Abstract

**Background:**

The amygdala has been proposed to be involved in the pathophysiology of pediatric and adult bipolar disorder (BD). The goal of this structural magnetic resonance imaging (sMRI) study was to investigate the morphometric characteristics of amygdala subnuclei in patients with pediatric bipolar disorder (PBD) compared to healthy controls (HCs). Simultaneously, we examined correlation between amygdala subnuclei volumes and cognitive dysfunction.

**Materials and Methods:**

We assessed 40 adolescent outpatients, diagnosed with manic or euthymic PBD according to the DSM-5 criteria for BD and 19 HCs. Cognitive functions were evaluated using a Stroop color-word test (SCWT), trail making test (TMT), visual reproduction immediate recall subtest (VR I), and digit span subtest (DST). Amygdala and its subnuclei structures were automated segmented using FreeSurfer software and the volumes of them were compared between groups and correlation with clinical and cognitive outcomes was conducted.

**Results:**

Manic patients exhibited significantly decreased volumes in the bilateral whole amygdala and its basal nucleus, cortico-amygdaloid transition (CAT), and accessory basal nucleus (ABN) compared with HCs. Euthymic patients had decreased volume in the bilateral ABN and left CAT. In addition, we found significant positive associations between VR I scores and the right whole amygdala and its bilateral basal, right lateral, and ABN volumes in the manic group.

**Conclusion:**

These findings support previous reports of smaller amygdala volumes and cognitive dysfunctions in PBD, and further mapping abnormalities to specific amygdala subnuclei. Correlation between basolateral volume and VR I of PBD may expand our understanding of neural abnormalities that could be targeted by treatment.

## Introduction

Pediatric bipolar disorder (PBD) is characterized by persistent influence dysregulation affects roughly 2% of youth under the age of 18 (1). Like bipolar disorder (BD) in adults, PBD is also characterized by recurring manic or hypomanic episodes and a depressive episode typically separated by periods of relative euthymia (2). Retrospective studies clearly indicate that pathology begins in childhood or adolescence for 50% to 66% of adults with BD (3). Early-onset BD may have worse outcomes including greater cognitive impairment (4), fewer days of euthymia (5), and suicide attempts (6). In these adolescents, the persistent affect dysregulation is often accompanied by increased risk of suicide (7) and severe cognitive impairment (8) leading to considerable deficits in memory, executive, processing speed, and verbal learning (5, 9). Therefore, it is important to have early objective biomarkers to detect cognitive impairment in order to minimize its negative impact on adolescent development. Such a biomarker would allow early and reliable identification and treatment of BD disorder-associated cognitive decline and shed light on the underlying mechanisms of BD development. However, no biomarkers for targeting or tracking the progression of BD in adolescents exist.

The amygdala is a key limbic region in modulating mood and emotions and is potentially involved in the cognitive and affective symptoms of BD (10). Neuropathologic and neuroimaging studies have implicated the amygdala as a central brain structure for processing emotions (11, 12), emotion-related aspects of behavior (13), attention (14), and memory (15). Converging evidence from neuroimaging studies has consistently implicated the dysfunction of the amygdala in the pathophysiology of BD. Kryza-Lacombe and colleagues (16) showed that youth and adult patients with BD had abnormal amygdala-temporo-parietal connectivity. Specifically, amygdala activation is inversely correlated with volume (17).

Numerous structural magnetic resonance imaging (MRI) studies indicate that smaller amygdala volumes may be an age-specific biomarker for BD. Decreased amygdala volumes in patients with PBD as compared with HCs have been reported in most studies (18–21). In contrast, studies of adults BD patients regarding the amygdala are markedly heterogeneous, with increased (22), not significantly different (23, 24), or decreased (25–27) amygdala volumes compared with HCs. These discrepancies likely reflect clinical and treatment heterogeneity. Some researchers speculate that amygdala volume is reduced at the onset of the disease and increases with age (26). A meta-analysis of the functional neural correlates of BD highlighted the amygdala as an area with unique developmental alterations in BD (28). Therefore, structural and functional amygdala abnormalities identified by neuroimaging may serve as useful disease and treatment response biomarker in BD.

The amygdala formation is commonly treated as a single entity in structural MRI; however, it is known to be comprised of multiple nuclei, each exhibiting different connectivities and cellular profiles (29). These subnuclei have diverse functions physiologically and have been shown in disease models of BD to react differentially to pathological mechanisms (30, 31). Due to the small size of the amygdala, few studies focused on volume changes of amygdala subnuclei in patients with PBD. Whether smaller amygdala volume has been localized to specific amygdala subnuclei in different clinical stages is unknown. With substantial advances in structural MRI tools, new amygdala segmentation algorithms have made it possible to label amygdala subnuclei and automatically provide volumetric information for each based on an *in vivo* atlas (32). Given this background, the goal of the current study was to compare amygdala and subnuclei volumes in a sample of manic or euthymic patients with PBD, and HCs. We hypothesized that the volumes of amygdala subnuclei would be smaller in patients with PBD than that of HCs. Moreover, we also hypothesized that worse cognitive abnormalities might be associated with these reduced amygdala subnuclei in patients with mania or euthymia.

## Materials and Methods

### Subjects

In this case-control study, all PBD patients were recruited from the Mental Health Institute of the Second Xiangya Hospital, Key Laboratory of Psychiatry and the Mental Health of Hunan Province of Central South University (Changsha, Hunan, China). We recruited forty right-handed patients with PBD across an age range of 12 to 18 years. All patients met DSM-5 criteria for BD (33), made up of two subgroups, mania (n = 20, 9 male/11 female), and euthymia (n = 20, 11 male/9 female). In addition, 19 right-handed age and sex-matched healthy control (HC) participants (7 male/12 female) were recruited from the local middle school *via* advertisements. All subjects were completed the Wechsler Abbreviated Scale of Intelligence as an overall measure of cognitive ability (34). General exclusion criteria were intellectual disability (IQ ≤ 80), left-handedness, substance abuse, history of seizures, history of electroconvulsive therapy (ECT), severe brain trauma, and MRI scan contraindications (e.g. metallic implants or claustrophobia).

All adolescents were assessed by the Schedule for Affective Disorders and Schizophrenia for School-Age Children-Present and Lifetime (KSADS-PL) (35) and the Washington University in St. Louis Kiddie Schedule for Affective Disorders and Schizophrenia (WASH-U-KSADS) (36). The K-SADS-PL for DSM-5 is a semi-structured diagnostic interview that assesses both current and lifetime diagnostic psychiatric episodes in children and adolescents. WASH-U-KSADS was developed specifically to target the assessment of prepubertal mania and hypomania and to assess the pattern of rapid cycling. Furthermore, severity of depression and mania were evaluated in all subjects by the Mood and Feelings Questionnaire (MFQ) (37), and Young Manic Rating Scale (YMRS) (38) respectively. The MFQ is a widely used screening measure of depressive symptomatology for children 8 to 18 years of age. The YMRS is an instrument used to assess the severity of mania in patients with a diagnosis of BD. The patients in the manic subgroup were required to have a YMRS score > 26 and MFQ score < 18, those in the euthymic subgroup were required to have had no episodes of illness for at least 1 month and YMRS score < 12 and MFQ score < 18 at the time of scanning. The inclusion criteria for HC included that the participants have no current or past DSM-5 psychiatric diagnosis, as confirmed by KSADS-PL, and no first- or second-degree family history of BD or other psychotic disorders.

This study protocol was approved by the University of Central South Institutional Review Board in compliance with the Declaration of Helsinki. After complete description of the study to adolescents and their parents, written informed consent and assent were obtained.

### Cognitive Function Test

To assess different aspects of cognitive functions, the cognitive estimate battery included the following: Stroop color-word test (SCWT), trail making test (TMT), visual reproduction immediate recall subtest (VR I), and digit span subtest (DST). The battery was administered by experienced clinical psychiatrists in a quiet environment. Below is a description of the various test procedures.

#### Stroop Color-word Test (SCWT)

The SCWT (39), measuring the ability to attention and response inhibition, included three tasks: word reading (SCWT-A), color naming (SCWT-B), and color interference reading (SCWT-C), each set contains 100 visual stimuli. SCWT-A is made up of the number of words that participants completed in 45 s. SCWT-B is made up of the number of symbols that subjects named correctly. SCWT-C is made up of the number of competing colors that participants read in 45 s.

#### Trail Making Test (TMT)

TMT is administered in the part A (TMT-A) and part B (TMT-B). TMT-A requires the subjects to draw a line between consecutive numbers (1–25) distributed on a piece of paper, and TMT-B requires the subjects to draw lines sequentially connecting 13 numbers (1–13) and 12 letters (A-L) distributed on a piece of paper. Numbers and letters are encircled and must be connected alternately. TMT score was the total times for subjects to complete the task. TMT-A reflect attention and processing speed, and part B reflects cognitive flexibility (40).

#### Visual Reproduction Immediate Recall Subtest (VR I)

The VR I was used to assessing visual memory, which check immediate recall and learning rate. Three pages of geometric designs are shown, one at a time. After viewing each graphic for 10 s, the participants are instructed to draw the graphics as accurately as possible from memory (41).

#### Digit Span Subtest (DST)

In the DST, the participant is asked to repeat the same sequence numbers back to the psychiatrists in forward order (DST-A) and in reverse order (DST-B). DST-A and DST-B were scored according to the longest series separately. DST-A was used to assess attention and DST-B measured working memory (34).

### MRI Acquisition and Analysis

All MRI scans were collected with a 3.0 T Siemens Trio system (Siemens, Erlangen, German) using a standard whole head coil. High-resolution anatomical scan was acquired using three-dimensional magnetization-prepared rapid acquisition gradient echo (3D MPRAGE) protocol with the following parameters: repetition time (TR) = 2300 ms, echo time (TE) = 2.98 ms, inversion time = 900 ms, thickness = 1 mm, gap = 0 mm, field of view (FOV) = 256 mm ×256 mm, matrix = 256× 256, flip angle = 9°.

**Figure 1:**
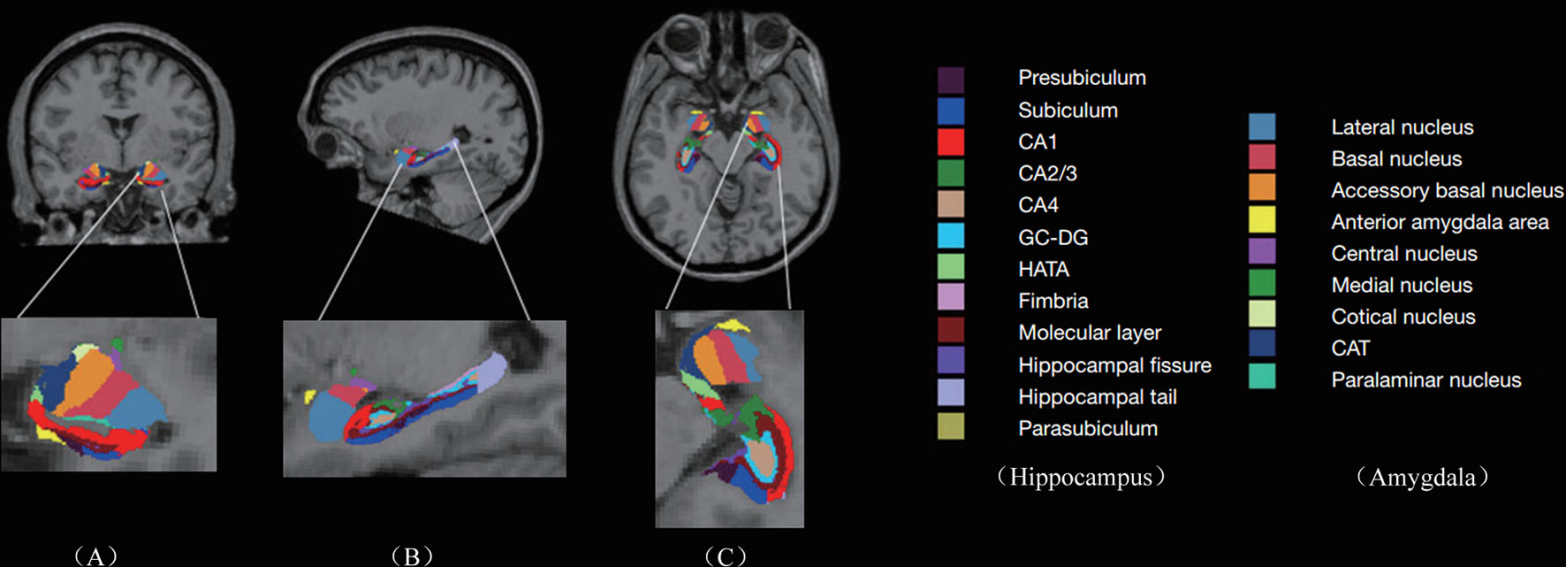
Subregions of hippocampus and amygdala: Columns **(A–C)** represent the image view of coronal, sagittal, and axial, respectively. The second row represents the enlarged subregions on the left. CA, cornus ammonis; GC-DG, granule cell layer of the dentate gyrus; HATA, hippocampal amygdala transition area; CAT, cortico-amygdaloid transition.

T1-weighted images were preprocessed by motion correction and brain extraction using FreeSurfer (version 6.0, https://surfer.nmr.mgh.harvard.edu). Each T1-weighted image was segmented into gray matter (GM), white matter (WM), and cerebrospinal fluid (CSF). Subsequently, the segmentation of subcortical structures was examined by a nonlinear warping atlas, yielding volumetric measures of Deep GM, including the thalamus, caudate, putamen, amygdala, hippocampus, pallidum, and accumbens. Furthermore, the amygdala subnuclei segmentation module, which is only present in the FreeSurfer dev version (ftp://surfer.nmr.mgh.harvard.edu/pub/dist/freesurfer/dev) was used to parcellate the hippocampus, amygdala, and thalamus subnuclei further, as shown in **Figure 1A**. A probabilistic atlas and a modified version of Van Leemput’s algorithm was applied on the segmentation of amygdala (32). In total the amygdala was divided into nine nuclei, including lateral, basal, accessory-basal nucleus (ABN), anterior-amygdaloid area (AAA), central, medial, cortical, cortico-amygdaloid transition (CAT), and paralaminar nucleus. Finally, using FreeSurfer’s native visualization toolbox, freeview, we visually inspected the segmentation of hippocampal/amygdala, as shown in **Figure 1**.

We used the Statistical Package for the Social Sciences (SPSS) for Windows, version 22.0 (SPSS Statistics, IBM, Armonk, NY, USA) to study the demographic, clinical, cognitive tests, and MRI data. The Shapiro-Wilk test was used to test for normality. Demographic, clinical, and cognitive test scores were evaluated using Pearson’s chi-square test, two-sample t-test or one-way ANOVA with a confidence interval of 95% where applicable. A general linear model (GLM) was used for group analysis of each subnuclei. The GLM was fitted with volume as the dependent variable, groups as the categorical predictor, and total intracranial volume (TIV), age, gender, and education were included as covariates. The indices with significant differences across the three groups were examined further by *post-hoc* differences. Multiple comparisons between groups were assessed using the Bonferroni method. We calculated the Spearman correlation coefficients between each subregion volume and each of the clinical and cognitive variables (onset age, illness duration, YMRS scores, and cognitive tests) for the PBD patients. In the correlation calculations, we regressed out the confounding factors of age, gender, education, and TIV. Spearman correlation results were corrected by false discovery rate (FDR) correction. Statistical significance for all tests was set at *p* < 0.05.

## Results

### Participants’ Characteristics and Cognitive Tests

Clinical, demographic, and cognitive test information was collected through self-report questionnaires and clinical interviews by trained psychiatrists. Demographic, clinical, cognitive tests, and medication regimen are summarized in **Table 1**. No group differences were observed in age (*F* = 3.118, *p* = 0.052), gender (chi-square = 1.301, *p* = 0.522), education (*F* = 2.153, p = 0.126), IQ (*F* = 1.503, *p* = 0.231) or MFQ (*F* = 0.429, *p* = 0.654). As expected, significant group differences were observed for YMRS (*F* = 356.537, *p* < 0.001). There were no significant differences in age of onset (t = 0.587, *p* = 0.561), illness duration (t = −1.687, *p* = 0.100), onset frequency (t = −0.983, *p* = 0.332), psychotic symptoms (chi-square = 0.902, *p* = 0.342), type of BD (chi-square = 0.125, *p* = 0.723) or familial BD history (chi-square = 0.476, *p* = 0.490) between two groups of PBD patients. In the mania subgroup, patients were taking the following medications: lithium (n = 8), valproate (n = 11), antipsychotics (n = 13), and antidepressants (n = 2). In the euthymic subgroup, patients were taking lithium (n = 8), valproate (n = 15), and antipsychotics (n = 15). There were significant differences in SCWT-A scores (*F* = 4.852, *p* = 0.011), SCWT-B scores (*F* = 8.023, *p* = 0.001), SCWT-C scores (*F* = 9.161, *p* < 0.001), TMT-A scores (*F* = 4.439, *p* = 0.016), VR I scores (*F* = 16.132, *p* < 0.001), and DST-B scores (*F* = 5.412, *p* = 0.007). Furthermore, the pairwise comparisons demonstrated apparent declines in SCWT, VR I, and DST-B scores in the two PBD groups (*p* < 0.05) compared with the HC group, as well as lower TMT-A scores in the manic patients (*p* < 0.05) compared with HC (**Table 1**). No significant difference was observed for TMT-B scores (*F* = 1.455, *p* = 0.242) or DST-A scores (*F* = 1.520, *p* = 0.228) among the 3 groups.

**Table 1:** Sample characteristics.

Characteristics		Manic-PBD (n=20)	Euthymic-PBD (n=20)	HC (n=19)	*F*/T/χ^2^	*p*	Pairwise comparisons (*p* value)		
							Mania vs. Euthymia	Mania vs. HC	Euthymia vs. HC
Gender (male/female)		9/11	11/9	7/12	1.301^#^	0.522	0.527	0.605	0.256
Age (years)		15.30 ± 1.81	15.60 ± 1.64	14.37 ± 1.30	3.118^&^	0.052	1.000	0.059	0.224
Education (years)		8.40 ± 1.76	8.70 ± 1.75	7.47 ± 2.22	2.153^&^	0.126	1.000	0.411	0.152
IQ		103.50 ± 10.67	108.60 ± 9.73	105.32 ± 7.51	1.503^&^	0.231	0.278	1.000	0.844
YMRS scores		34.30 ± 6.44	5.50 ± 1.70	3.63 ± 2.06	356.537^&^	<0.001^***^	<0.001***	<0.001***	0.467
MFQ scores		7.15 ± 2.62	6.65 ± 4.38	6.11 ± 3.33	0.429^&^	0.654	1.000	1.000	1.000
Onset age (year)		14.05 ± 1.73	13.70 ± 2.03	–	0.587^^^	0.561	–	–	–
Illness duration (months)		15.90 ± 12.96	24.15 ± 17.62	–	−1.687^^^	0.100	–	–	–
Onset frequency		3.10 ± 1.68	4.80 ± 7.55	–	−0.983^^^	0.332	–	–	–
The first episode bipolar disorder (mania/depression)		9/11	7/13	–	0.417^#^	0.519	–	–	–
Acute or delayed onset (acute/delayed)		10/10	11/9	–	0. 010^#^	0.752	–	–	–
Psychotic symptoms(yes/no)		9/11	12/8	–	0.902^#^	0.342	–	–	–
BP-I/BP-II		15/5	14/6	–	0.125^#^	0.723	–	–	–
Familial BD history(yes/no)		7/13	5/15	–	0.476^#^	0.490	–	–	–
Medications	Lithium	8	8	–	–	–	–	–	
	Valproate	11	13	–	–	–	–	–	
	Atypical antipsychotics	13	15	–	–	–	–	–	
	Antidepressants	2	–	–	–	–	–	–	
SCWT-A		53.35 ± 15.98	54.35 ± 13.53	66.00 ± 12.26	4.852^&^	0.011^*^	1.000	0.020*	0.037*
SCWT-B		69.80 ± 19.56	71.80 ± 15.16	87.79 ± 9.08	8.023^&^	0.001^**^	1.000	0.002**	0.006**
SCWT-C		29.65 ± 7.36	31.85 ± 9.12	40.74 ± 9.43	9.161^&^	<0.001^***^	1.000	<0.001***	0.006**
TMT-A		40.35 ± 12.31	38.15 ± 12.80	29.74 ± 9.63	4.439^&^	0.016^*^	1.000	0.019*	0.086
TMT-B		88.90 ± 30.64	101.95 ± 50.89	80.61 ± 31.27	1.455^&^	0.242	0.844	1.000	0.294
VRT		8.45 ± 3.41	10.30 ± 2.60	13.21 ± 1.48	16.132^&^	<0.001^***^	0.091	<0.001***	0.003**
DST-A		8.20 ± 1.51	8.65 ± 1.66	9.00 ± 1.05	1.520^&^	0.228	0.980	0.264	1.000
DST-B		4.45 ± 1.15	4.75 ± 1.62	5.95 ± 1.68	5.412^&^	0.007^**^	1.000	0.009**	0.047*

Data are presented as mean ± standard deviation. ^#^Pearson chi-square test; ^&^one-way ANOVA (analysis of variance); ^^^Independent-sample t-test. The pairwise comparisons between groups using Bonferroni method. *p < 0.05, **p < 0.01, ***p < 0.001.

IQ, intelligence quotient; YMRS, Young Manic Rating Scale; MFQ, Mood and Feelings Questionnaire; BP-I, bipolar disorder type I; BP-II, bipolar disorder type II; SCWT, Stroop color-word test; TMT, trail making test; VR I, visual reproduction immediate recall subtest; DST, digit span test.

### Subnuclei Volume Analysis

**Figure 2:**
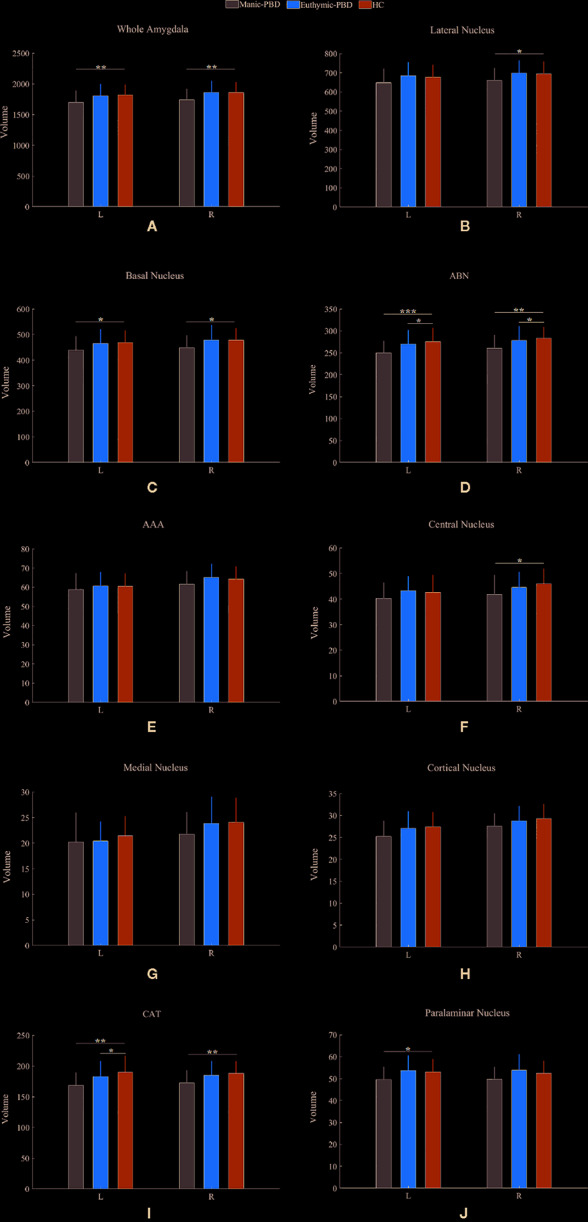
Pairwise comparison of volumes in the manic PBD group, euthymic PBD group, and healthy control group (**A**,Whole Amygdala volume; **B**, Lateral Nucleus volume; **C**, Basal Nucleus volume; **D**, ABN volume; **E**, AAA volume; **F**, Central Nucleus volume; **G**, Medial Nucleus volume; **H**, Cortical Nucleus volume; **I**, CAT volume; **J**, Paralaminar Nucleus volume). The Y-axis represents the mean volume of amygdala and its subnuclei in each group. Unit: mm^3^. *p < 0.05; **p < 0.01. L, left amygdala; R, right amygdala; ABN, accessory basal nucleus; AAA, anterior amygdaloid area; CAT, cortico-amygdaloid transition.

**Table 2** summarizes the statistical analysis for the volume of the amygdala subnuclei. There were significant differences in the bilateral whole amygdala, basal nucleus, ABN, and CAT, left cortical nucleus, left paralaminar nucleus, and right central nucleus among the three groups (*p* < 0.05). Histograms in **Figure 2** demonstrate post-hoc pairwise comparisons on amygdala subnuclei volumes. **Table 2** lists the statistical results of *post-hoc* pairwise comparisons in subnuclei with significant differences among the three groups. The strongest effects for bilateral whole amygdala, ABN, and CAT volume decrease were seen in manic patients (*p* < 0.01). In addition, euthymic PBD group exhibited deceased bilateral ABN and left CAT volumes compared with HCs (*p* < 0.05).

**Table 2:** The difference among the three groups in the amygdala and subnuclei.

Regions		Manic-PBD (n = 20)	Euthymic-PBD (n = 20)	HC (n = 19)	*F* ^#^	*p*	Pairwise comparisons (*p* value)		
							Mania vs. Euthymia	Mania vs. HC	Euthymia vs. HC
Left	Whole amygdala	1699.614 ± 193.038	1805.293 ± 195.487	1815.260 ± 174.344	4.669	0.014*	0.145	0.004**	0.121
	Lateral nucleus	647.823 ± 72.738	683.317 ± 70.861	676.520 ± 65.766	1.776	0.180	0.274	0.068	0.447
	Basal nucleus	439.095 ± 54.010	464.978 ± 55.652	468.220 ± 47.291	3.274	0.046*	0.263	0.013*	0.161
	ABN	250.118 ± 27.183	269.462 ± 32.278	275.435 ± 31.274	8.345	0.001**	0.075	<0.001***	0.028*
	AAA	58.851 ± 8.472	60.574 ± 7.341	60.381 ± 6.779	0.113	0.893	0.901	0.644	0.739
	Central nucleus	40.210 ± 6.246	43.291 ± 5.649	42.654 ± 6.794	1.109	0.368	0.306	0.183	0.737
	Medial nucleus	20.219 ± 5.779	20.390 ± 3.818	21.452 ± 3.815	0.811	0.450	0.658	0.396	0.215
	Cortical nucleus	25.228 ± 3.544	27.040 ± 3.934	27.368 ± 3.429	3.288	0.045*	0.324	0.014	0.127
	CAT	168.509 ± 21.032	182.552 ± 25.746	190.128 ± 26.655	6.470	0.003**	0.123	0.001**	0.048*
	Paralaminar nucleus	49.561 ± 5.853	53.688 ± 6.877	53.101 ± 5.820	3.256	0.047*	0.058	0.022*	0.642
Right	Whole amygdala	1741.491 ± 175.730	1856.749 ± 192.371	1859.253 ± 168.184	5.184	0.009**	0.050	0.003**	0.243
	Lateral nucleus	658.386 ± 65.822	699.189 ± 64.786	692.813 ± 65.799	2.860	0.066	0.057	0.038*	0.814
	Basal nucleus	448.180 ± 48.673	478.357 ± 59.766	478.559 ± 45.965	3.508	0.037*	0.114	0.012*	0.313
	ABN	260.065 ± 30.681	278.116 ± 33.518	283.288 ± 25.640	6.825	0.002**	0.115	0.001**	0.042*
	AAA	61.595 ± 6.781	65.147 ± 7.057	64.180 ± 6.641	1.151	0.324	0.168	0.25	0.851
	Central nucleus	41.861 ± 7.571	44.650 ± 5.929	46.013 ± 5.886	3.226	0.048*	0.420	0.015*	0.099
	Medial nucleus	21.761 ± 4.291	23.858 ± 5.181	24.000 ± 4.893	1.203	0.308	0.386	0.130	0.504
	Cortical nucleus	27.501 ± 2.979	28.732 ± 3.444	29.276 ± 3.349	1.730	0.187	0.560	0.072	0.222
	CAT	172.367 ± 20.692	184.866 ± 23.368	188.620 ± 19.389	4.988	0.010*	0.125	0.003**	0.115
	Paralaminar nucleus	49.776 ± 5.556	53.834 ± 7.309	52.502 ± 5.646	2.520	0.090	0.065	0.057	0.911

Data are presented as mean ± standard deviation. The pairwise comparisons between groups using Bonferroni method. ^#^ANCOVAs (Analysis of covariance). nit: mm^3^. *p < 0.05, **p < 0.01, ***p < 0.001.

ABN, accessory basal nucleus; AAA, anterior amygdaloid area; CAT, cortico-amygdaloid transition.

### Correlation Analysis

**Figure 3:**
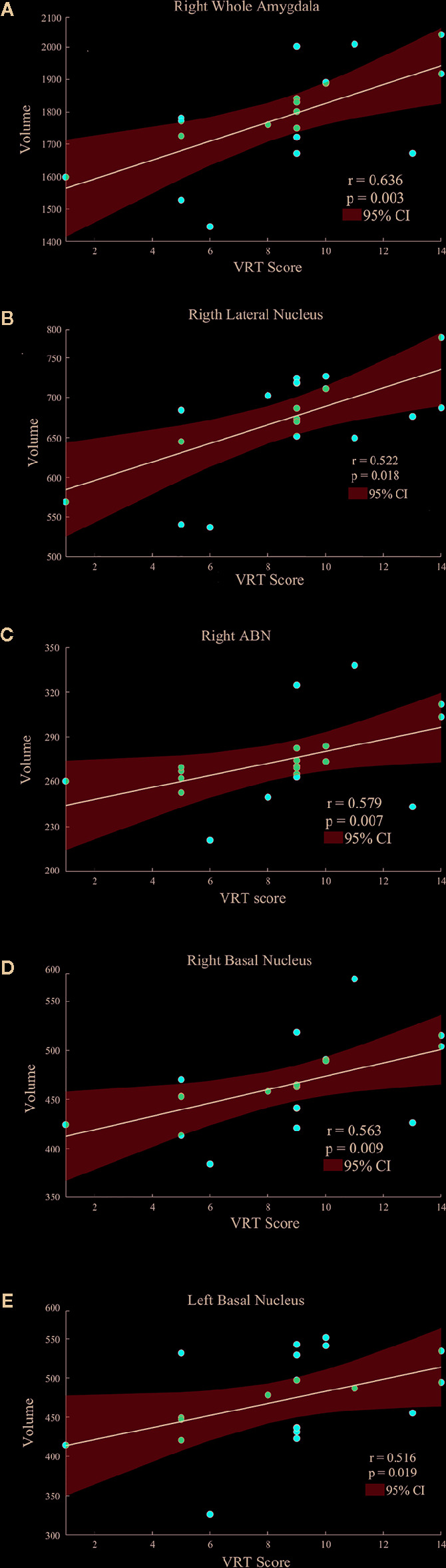
Scatter plots showing the relationships between the amygdala and subnuclei volumes and VR I score in manic patients group (**A**, Right Whole Amygdala; **B**, Right Lateral Nucleus; **C**, Right ABN; **D**, Right Basal Nucleus; **E**, Left Basal Nucleus). ABN, accessory basal nucleus; VR I, visual reproduction immediate recall subtest.

Age, gender, and years of education were not significantly correlated with amygdala morphology within the HCs and PBD groups. In the manic PBD group, VR I score was found to be positively correlated with right whole amygdala, bilateral basal nucleus, right lateral nucleus, and right ABN volume (*p* < 0.05; **Figure 3**). The euthymic PBD group had no significant correlation between the subnuclei volume and any of the clinical and cognitive characteristics.

## Discussion

To the best of our knowledge, this cross-sectional study is the first work utilizing automated neuroanatomical quantification (FreeSurfer) to evaluate amygdala and subnuclei volumetric differences in PBD patients. The main finding of the present study was the significant differences in the basal nucleus, ABN, and CAT volumes between PBD patients and HCs. Unexpectedly, amygdala and subnuclei volumes between manic and euthymic patient group were indistinguishable for all structures examined. In addition, this study detected that PBD patients have significant differences in SCWT, TMT-A, VR I, and DST-B compared to HCs. And in the manic PBD group, VR I score was found to be positively correlated with right whole amygdala, bilateral basal nucleus, right lateral nucleus, and right ABN volume.

Neuroimaging studies in PBD have so far supported the key role of amygdala. Alterations in amygdala volumes have been associated with measures of illness duration and disease progression in PBD (20). Consistent with previous finding, the present study specifically found a mean volume reduction of 6.4% in left amygdala, and 7.8% in right amygdala in manic PBD patients compared with that of the HCs, with no significant difference in euthymic PBD patients (**Figure 2A**). Furthermore, the mania and euthymia group showed differences in right whole amygdala volume, but it did not attain the statistical significance (*p* = 0.050). It is worth noting that little evidence of amygdala volumetric alterations was reported in young subjects with schizophrenia (SZ) or other psychotic disorders, indicating that alterations may be specific to BD (42). Post mortem studies had reported the amygdala as a common site for senile plaques and neurofibrillary tangles in Alzheimer’s disease (AD) and mild cognitive impairment (43). McGaugh emphasized that the amygdala is critically related with memory consolidation by intermediating the impacts of epinephrine and glucocorticoids and regulating the activities of striatum and hippocampus (44). In healthy individuals, amygdala volume has no connection with memory function, whereas in BD patients, larger amygdala volume was predictive of integrated memory function (24). Consistent with our study, we found that right whole amygdala volume was predictive of cognitive performance in manic group, correlating positively with better immediate recall memory for manic PBD patients (**Figure 3A**).

We determined *in vivo* localization of the volumetric difference within the amygdala. The most affected subnuclei were the bilateral ABN and left CAT in all PBD patients (**Figures 2D, I**), and volume changes in the bilateral basal, left paralaminar nucleus, and right CAT, lateral, and central nucleus were apparent only in manic patients (**Figures 2B, C, I, J**). Originating in the anteromedial temporal lobe, the amygdalofugal tract passes through the basal, lateral, and central amygdala nucleus toward the midline (45), which is believed to apply downstream control over hypothalamus and septal nuclei, affecting threat reactivity and memory (46). The central nucleus is a key output area for expressing innate emotional responses and associated physiological responses, and it connects brainstem controlling specific behaviors and physiological responses. The basal nucleus is another important region of output connecting with the central nucleus; and the striatal areas are related with controlling of instrumental behaviors. In addition, connections from the basal amygdala to the striatum are involved in controlling actions. The lateral nucleus in is believed to tie cortical areas account for processing sensory stimuli with structures responsible for eliciting emotional responses to these stimuli. Therefore, we suggested that the ABN and CAT may serve as early image markers for differentiating patients with PBD from HCs and the volume of basal, lateral, and central nucleus for targeting or tracking the progression of illness in adolescents BD.

The amygdala can be generally partitioned into two major subdivisions: the basolateral (BLA), and centrocorticomedial. The ABN, basal, and lateral nucleus constitute the BLA complex (25, 47), which comprises 69% of the total amygdala volume in humans. The BLA group is thought to represent an integration center for coordinating inputs from certain cortical and subcortical regions, including the prefrontal cortex (PFC), hippocampus, thalamus, and visual cortices; the BLA is involved in learning and memory (48). The cortical, medial, and central nucleus belong to the centrocorticomedial group (49), which has been suggested to receive astrictive information from the medial PFC and BLA, thereby serve as the pathway to generate behavioral, motor, and autonomic emotional responses (50). The results showed that the decreased volume of amygdala subnuclei in PBD patients were mainly concentrated in the BLA.

In psychiatric disorders, neurocognitive impairments are prevalent and have been associated with poor outcome (51). The cognitive tests used in this study cover a broad range of cognitive abilities, including attentional capacity measured with DST-A and TMT-A; processing speed measured with TMT-A, SCWT-A (color naming), SCWT-B (word reading); working memory/mental tracking measured with DST-B; visual memory measured with VR I (immediate recall); self-regulation/self-monitoring measured with SCWT-C (inhibition); and cognitive flexibility measured with TMT-B (Number-Letter Switching) (52). In this study, SCWT, TMT-A, VR I, and DST-B completion scores differed significantly between the patients and HCs. The results provide evidence that manic and euthymic patients with PBD have significant cognitive impairment, specifically in processing speed, executive function, visual learning, and working memory.

The Spearman correlation of this study indicated that amygdala subnuclei association with VR I scores are primarily in the right BLA (**Figures 3B–D**). Except for immediate recall memory, VR is also related to visual-perceptual-motor and nonverbal reasoning memory. VR has a widely of clinical and research utility, often employed in AD (53), posttraumatic stress disorder (54), major depressive Disorder (55), autism spectrum disorder (56). Troster et al. (53) found that VR had excellent sensitivity and specificity in differentiating patients with AD from HCs. Mak and colleagues (55) found that unipolar and bipolar patients with depression could be distinguished by a relatively intact cognitive profile, including TMT and VR. As a result, the right BLA group may serve as early imaging markers for the visual memory dysfunction of manic PBD patients. The relationship between scores in VR I, the right whole amygdala and BLA volumes may evoke a long-existing theory of left-right dissociation of memory systems. This controversial hypothesis suggests that the left amygdala may be responsible for verbal information, whereas visuospatial data may be stored within the right amygdala (57).

There are several limitations in our study. Medication (lithium and other mood stabilizers like valproic acid) could influence the amygdala and subnuclei volumes of patients with PBD. In this study, most of the adolescent patients were taking more than one drug, so we could not rule out drug effects on the results. Moreover, this was a cross-sectional study. Future studies would benefit from longitudinal monitoring to determine whether discrete syndromes have different patterns of amygdala and subnuclei volume changes during an individual’s clinical progression.

In conclusion, we used a novel, automated approach to segment and evaluate differences in amygdala and subnuclei volumes in patients with PBD. Together our neuroimaging and cognitive function findings suggest that the volumes of amygdala subnuclei were smaller in manic and euthymic patients with PBD than that of HCs, especially the ABN and CAT. In addition, visual memory abnormalities might be associated with right whole amygdala, bilateral basal nucleus, right lateral nucleus, and right ABN volume reductions in patients with manic. Moreover, our findings suggest that smaller BLA group volumes may be an early marker of PBD progression toward weaker cognitive function.

## Data Availability Statement

All datasets generated for this study are included in the article/supplementary material.

## Ethics Statement

The studies involving human participants were reviewed and approved by University of Central South Institutional Review Board. Written informed consent to participate in this study was provided by the participants’ legal guardian/next of kin. Written informed consent was obtained from the individual(s), and minor(s)’ legal guardian/next of kin, for the publication of any potentially identifiable images or data included in this article.

## Author Contributions

JQ, LS, and GL designed the study. WG and LS acquired the data. DC, YG, and WC processed all neuroimaging data. DC and JQ performed all statistical analyses. DC wrote the article, which all authors reviewed. All authors contributed to the article and approved the submitted version.

## Funding

We are grateful for support from the Fundamental Research Funds for the Central Universities (No. 3332018159 to DC), Funds of the National Natural Science Foundation of China (81371531 to QJ, 81901730 to WC), Key Project of Scientific Research of “12th Five-Year Plan” in Medical Research of the Army (BWS11J063 to GL), Academic Promotion Programme of Shandong First Medical University (No. 2019QL009), and JQ was supported by the Taishan Scholars Program of Shandong Province (No. TS201712065).

## Conflict of Interest

The authors declare that the research was conducted in the absence of any commercial or financial relationships that could be construed as a potential conflict of interest.
